# Adipose Tissue Extracellular Matrix Remodeling in Response to Dietary Patterns and Exercise: Molecular Landscape, Mechanistic Insights, and Therapeutic Approaches

**DOI:** 10.3390/biology11050765

**Published:** 2022-05-17

**Authors:** Ivo Vieira de Sousa Neto, João Luiz Quagliotti Durigan, Adelino Sanchez Ramos da Silva, Rita de Cássia Marqueti

**Affiliations:** 1 Molecular Analysis Laboratory, Faculty of Ceilândia, Universidade de Brasília, Brasília 70910-900, Brazil; marqueti@gmail.com or; 2 Graduate Program in Rehabilitation Sciences, Universidade de Brasília, Brasília 70910-900, Brazil; joaodurigan@gmail.com; 3 Graduate Program in Rehabilitation and Functional Performance, Ribeirão Preto Medical School, Universidade de São Paulo, Ribeirão Preto 14040-900, Brazil; adelinosanchez@usp.br; 4 School of Physical Education and Sport of Ribeirão Preto, Universidade de São Paulo, Ribeirão Preto 14040-900, Brazil; 5 Graduate Program in Health Sciences and Technology, Universidade de Brasília, Brasília 70910-900, Brazil

**Keywords:** metabolic organ, extracellular compartment, physical activity, nutrition

## Abstract

**Simple Summary:**

Adipose tissue is considered a metabolic organ that adjusts overall energy homeostasis and critical hormones to the body’s needs. In conditions of caloric intake surpassing energy expenditure, lipid accumulation occurs with constant extracellular matrix deposition. Excess lipids and adipocyte hypertrophy may reduce extracellular matrix flexibility in conjunction with hypoxia and inflammation. These processes induce the development of adipose tissue fibrosis and correlated metabolic dysfunctions, such as insulin resistance. With the increasing rate of chronic diseases worldwide, it is essential to generate a more precise knowledge of fibrotic processes, as well as to create optimal models to study potential therapies to combat the harmful effects of extracellular matrix deposition. In this work, we focused on the physiological processes in the remodeling of adipose tissue fibrosis, along with their relevance to clinical indications. Furthermore, we emphasize understanding how lifestyle can alleviate adipocyte dysfunction. Several studies showed that a nutritionally balanced diet combined with exercise is a remarkable potential strategy for lipolytic activity, preventing rapid extracellular matrix expansion in parallel with insulin and glucose action improvements. Thus, the emerging beneficial role of exercise training and low-calorie diet on adipose tissue ECM remodeling is a topic that deserves attention from health professionals.

**Abstract:**

The extracellular matrix (ECM) is a 3-dimensional network of molecules that play a central role in differentiation, migration, and survival for maintaining normal homeostasis. It seems that ECM remodeling is required for adipose tissue expansion. Despite evidence indicating that ECM is an essential component of tissue physiology, adipose tissue ECM has received limited attention. Hence, there is great interest in approaches to neutralize the harmful effects of ECM enlargement. This review compiles and discusses the current literature on adipose tissue ECM remodeling in response to different dietary patterns and exercise training. High-calorie diets result in substantial adipose tissue ECM remodeling, which in turn could lead to fibrosis (excess deposition of collagens, elastin, and fibronectin), inflammation, and the onset of metabolic dysfunction. However, combining a nutritionally balanced diet with exercise is a remarkable potential strategy for lipolytic activity, preventing rapid ECM expansion in different adipose tissue depots. Despite the distinct exercise modalities (aerobic or resistance exercise) reversing adipose tissue fibrosis in animal models, the beneficial effect on humans remains controversial. Defining molecular pathways and specific mechanisms that mediate the positive effects on adipose tissue, ECM is essential in developing optimized interventions to improve health and clinical outcomes.

## 1. Introduction

Adipose tissue is considered a specific connective tissue consisting of lipid-rich cells. It provides for the body’s survival by storing or dispensing energy for primary metabolism, as well as modulating thermogenesis immune and endocrine responses, which are crucial for overall homeostasis [1]. The complex role of the adipose tissue in metabolism places specific demands on cell morphology and adipocyte arrangement [1]. The dynamic structure of the extracellular matrix (ECM) is crucial for their functioning. The ECM controls structural support, mechanical stability, cell function, and adipocyte development [2,3]. This ECM plasticity of adipose tissue ensures that physiologic stimuli modulate adhesion, migration, and survival and subsequently alter cell phenotype [4].

ECM is formed principally in adipose tissue by collagens (I, VII), fibronectin, elastin, glycosaminoglycan (GAG), and a small quantity of laminin [5,6,7,8]. ECM remodeling is necessary to provide enough space for adipocyte hypertrophy and precursor cell hyperplasia [6]. Pathological conditions and signs are characterized by excessive amounts of ECM components, leading to dysfunctional remodeling and ECM stiffness, which can ultimately cause severe disturbances in tissue function [9]. Excessive ECM production in adipose tissue (i.e., fibrosis) is a characteristic of the unhealthy phenotype found in non-alcoholic steatohepatitis development, inflammatory conditions, insulin resistance, and breast cancer [10,11,12,13,14]. Recent evidence indicated that fibrosis is strongly associated with obesity, metabolic dysfunction, and difficulty in weight loss, suggesting that the ECM microenvironment may increase disease progression [6,9,15]. Presently, there is great concern in investigating approaches to attenuate the harmful effects of ECM enlargement.

In combination with a nutritionally balanced diet, exercise training is a remarkable potential strategy for lipolytic activity, preventing rapid ECM expansion and attenuating inflammatory conditions in parallel with insulin action improvements [7]. Some evidence showed that exercise training might play an essential role in adipose tissue ECM remodeling, previously altered by high-fat (HF) diets in rodents [7,16], promoting a decrease in the collagen deposition, metalloproteinase (MMP) activity, and gene expression of ECM components, which are essential for maintaining metabolic health. In contrast, the impact of controlled exercise on adipose tissue ECM in obese humans is scarce and controversial, probably due to the lack of precise control of dietary intake and demographic factors [17,18]. Increasingly in-depth analysis of the ECM will improve knowledge of their adipose tissue plasticity and encourage novel therapeutic strategies, which might be of particular interest to health professionals. However, the clinical relevance of adipose tissue ECM is scattered throughout the literature.

Starting from the background outlined above, we compile and discuss the current literature on adipose tissue ECM remodeling in response to dietary patterns and exercise training in this integrative review. We first provide the historical framework of the field, then focus on revisiting the advances in the understanding of adipose tissue biology, ECM composition, and function. In the second part, we update the importance of the morphological, cellular, and molecular ECM modification pathways inherent to different diets and exercise protocols, ending with a perspective on how this knowledge may be translated into therapeutic approaches for chronic diseases. We presented an impartial description of the effects of exercise training and dietary interventions, such as obesity and high-fat diet in rodent studies, providing new insights into the biological meaning of the phenomenon. Moreover, we discuss vital mechanisms, major research tracks, and critical gaps in this emerging area, providing essential discussion and valuable conclusions to the adipose tissue biology field. These reports provide clinically useful information and help design effective nutritional and exercise interventions. Our remarks have high relevance in physiology, nutrition, and sports medicine.

## 2. Adipose Tissue Complexity and Critical Functions

**Figure 1:**
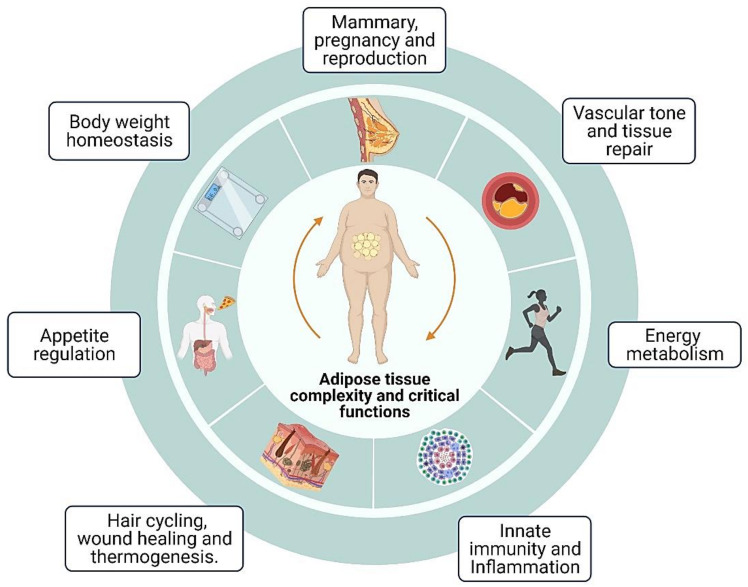
Adipose tissue in mammals participates in various body biological functions. Representation of the main physiological process involved. Biorender web-based software was used to create the figure (License Number LZ23TBWGZ1).

Adipose tissue is a connective tissue consisting of lipid-rich cells called adipocytes. This tissue also contains fibroblasts, vascular endothelial cells, immune cells, and primarily extracellular matrix (ECM) [2]. Adipose tissue makes up around 20–25% of the total body weight in healthy persons. The most important functions of adipose tissue are mechanical cushioning, thermogenic capacity, and energy storage in triglycerides inside their cytoplasmic lipid droplets [1]. Mainly, multicellular organisms have evolved specialized cells such as adipocytes to store excess nutrients as lipids because this macro biomolecule has higher calories when compared to other essential nutrients [19]. The energy storage obtained during food abundance is a compensatory physiological process that enhances survival during extended periods of food scarcity, representing a protective mechanism for the maintenance of metabolic homeostasis [20]. Adipose tissue is characterized as complex and multi-faceted organ quantitatively important for energy storing, hormone production, lactation, thermal isolation, and the endocrine profile of the body, besides protecting diverse organs and holding the cells in place. This tissue complexity and main functions are reported in Figure 1.

For several decades, adipose tissue has been characterized as a passive fuel reservoir. However, it is currently considered a critical endocrine organ, which secretes key bioactive factors, such as metabolites, hormones, growth factors, and anti/pro-inflammatory factors, exerting multiple impacts on the regulation of systemic energy [21]. The most important molecules include adiponectin, leptin, resistin, visfatin, chemerin, cytokines, and chemokine [22]. These molecules in blood circulation deliver information to other metabolic organs, including the liver, pancreas, muscle, and brain. The autocrine/paracrine interplay of local factors and molecular network within the adipose tissue influences other tissues on the endocrine level [1]. The discovery of adipokines in the last years revealed tissue secretome as a central point in the crosstalk between physiological systems [22].

In mammals, adipose tissue is located beneath the skin, around internal organs (visceral fat), in bone marrow (yellow bone marrow), between muscles, and in the breast [22]. Abdominal fat has a unique metabolic profile, and it has a high impact on insulin resistance [23]. Parietal fat plays a vital role in thermoregulation, while visceral fat supports internal organs, protecting them from mechanical forces [24]. Adipose tissue is observed in specific sites, which are referred to as depots [24]. Emerging evidence suggests that different depots have distinct molecular and biochemical profiles, which change the critical function for remodeling in response to excess or deprivation of nutrients, as well as exercise training [25,26].

**Figure 2:**
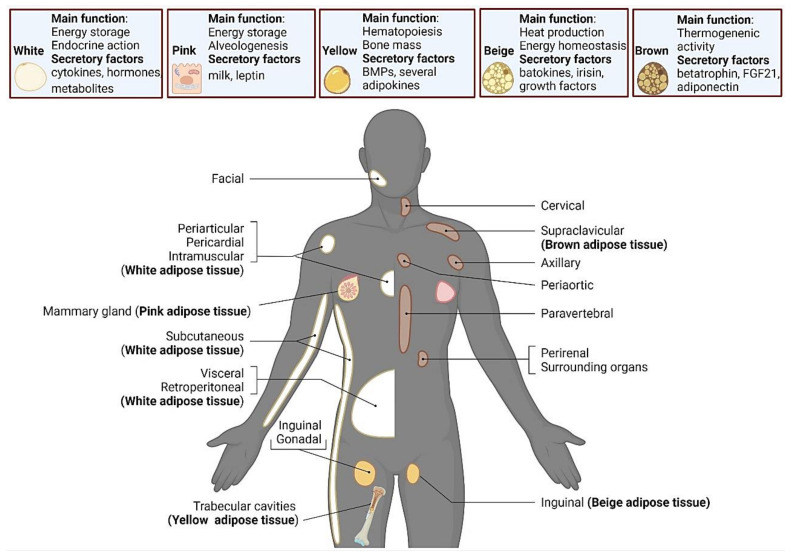
The distinct functions and localizations of white, pink, yellow, beige, and brown adipocytes. Biorender web-based software was used to create the figure (License Number QR23TBWNJ2).

Based on morphology, there are five major adipose tissue types: white, pink, beige, brown, and yellow adipose tissue [27]. White adipocytes can store and release energy in the form of significant lipids as a unilocular droplet in the cytoplasm [22]. Consequently, multiple inflammatory processes, robust ECM deposition, high expression of collagens and enzymes were observed to be involved in white adipose tissue remodeling [28]. Adipocytes acquire a pink color in pregnancy and lactation in alveolar cells [29]. Brown adipocytes are categorized with high mitochondrial content and several tiny lipid droplets in the cytoplasm. Brown adipocytes also secrete hormones and growth factors (Fibroblast growth factor 21 and betatrophin). These adipocytes need an oxidative metabolism and contain adrenergic nerve fibers, as well as high levels of fluorodeoxyglucose [29]. Cell–extracellular matrix connections have been demonstrated to induce beige and brown adipose formation [30]. Cells cultured on complex ECM displayed an upregulation of uncoupling protein 1 (UCP1) expression, a primary marker of beige/brown adipocytes, while diverse ECM proteins were reduced [30]. Subsequently, beige adipocytes are distinguished by multilocular lipid droplets and high mitochondrial numbers, in addition to playing a central role in uncoupled respiration, through key uncoupling proteins described in the organelles [27,31]. Moreover, it is also an important location for thermogenesis, and it is associated with lipolysis upgrade [9]. The unique gene expression signature of beige adipocytes may reflect the density of multilocular adipocytes and their level of differentiation [29]. Finally, the color yellow was designated to combine characteristics of white and brown with initial localization in trabecular cavities, regulating hematopoiesis and bone mass [29]. In lean subjects, yellow fat depots correspond to around 10% of total fat mass. Despite exhibiting unilocular morphology, it appears to be resistant to triglyceride storage compared to white adipose tissue [29]. Adipose tissue colors, localization, and secretory factors are displayed in Figure 2.

## 3. Adipose Tissue Plasticity and Chronic Diseases

Excessive ectopic lipid accumulation in organs is associated with cardiovascular diseases, overweight, and diabetes [32]. Adipose tissue dysfunction is central to obesity-related metabolic development and lipodystrophy. There is an imbalance between fatty acid oxidation and storage in this condition, which generates toxic lipid intermediates synthesis, including diacylglycerol and ceramides [32]. These excess compounds are associated with hypertrophic adipocytes, hypoxia, and chronic low-grade inflammation, all related to tissue and systemic insulin resistance [9]. Of note, ECM remodeling is a prerequisite to providing enough space for the new cell through adipogenesis.

Adipogenesis is a complex phenomenon in which precursor stem cells differentiate into lipid-laden adipocytes [33]. This differentiation is described by sequential gene expression adjustments that produce a mature adipocyte phenotype [34]. These alterations involve the appearance of early, intermediate, and late mRNA targets. The development arises from three steps: growth, mitotic clonal expansion, and terminal cell differentiation [34]. This first step is governed by synergistic actions of transcription factors such as peroxisome proliferator-activated receptor γ (PPARγ) and the CCAAT/enhancer-binding proteins (C/EBPs) [33]. Subsequently, fatty acid-binding protein 4 (FABP4), fatty acid synthase (FAS), and sterol regulatory element-binding protein 1 (SREBP1) are considered the critical early regulators responsible for the formation of mature adipocytes [35].

Chronic diseases are highlighted as pro-inflammatory conditions in which adipose tissue is considered an immune organ at the crossway between immunity and metabolism [36]. Adipose tissue includes a variety of immune cells, either adaptive (B and T lymphocytes) or innate (macrophages, neutrophils, and myeloid-derived suppressor cells), which contribute to the increase in pro-inflammatory cytokine levels [37,38]. Obese adipose tissue releases mainly pro-inflammatory cytokines (i.e., tumor necrosis factor-alpha (TNF-α,) interleukin (IL)-6, IL1b, and interferon-gamma (IFN-γ)) through inflammatory pathway [39] stimulations, including the c-Jun N-terminal kinase (JNK) pathway and I-kappa B kinase β (IKKβ)/NFκB [40]. At the same time, anti-inflammatory adipokines such as adiponectin, interleukin (IL)-10, IL-4, IL-13, IL-1 receptor antagonist (IL-1Ra) are under-expressed. Most significantly, the excess ECM deposition in adipose tissues was noted along with tissue damage, described by the neutrophils, lymphocytes, and mainly macrophages infiltration.

Macrophage infiltration and inflammation-related gene expression precede insulin resistance in adipose tissue and appear to be a primary feature of obesity [19]. Additionally, adipose tissue macrophages are also phenotypically altered during metabolic diseases to increase the number of cells. In obese individuals, adipose tissue predominantly contains pro-inflammatory M1 macrophages compared to anti-inflammatory M2 [41]. Activated M1 is a prominent source of pro-inflammatory cytokines [41,42], which can block insulin action in adipocytes through autocrine/paracrine signaling affecting insulin overall homeostasis via endocrine signaling pathways [19]. Therefore, macrophage recruitment and pro-inflammatory polarization are necessary for insulin resistance development in adipose tissue during metabolic diseases.

It is proposed that ECM enlargement might contribute to type-2 diabetes and progression toward metabolic syndrome [3]. The insulin sensitivity as a pathogenic factor represents a vital target driving current and future treatments. The ECM adipose tissue expandability leads to cell death and insulin resistance, providing one explanation for obesity-driven insulin resistance [3,6]. Evidence suggests that communications between adipocyte membranes and ECM possibly will trigger the β1 integrin and extracellular signal-regulated kinase signaling pathway and consequently affect insulin sensitivity by changing the arrangement of membrane caveolae where insulin receptors are contained [43].

The adipose tissue ECM from subjects with diabetes mellitus impairs glucose uptake and decreased lipolysis in adipocytes, which suggests that these cells can modulated hyperglycemia/hyperinsulinemia [44]. The transition from normoglycemia to resistance insulin in patients with diabetes was associated with enhanced collagen deposition and lumican in the omental adipose tissue [45]. Indeed, it was observed that obese individuals with insulin resistance exhibited increased pericellular fibrosis in omental adipocytes when compared to normoglycemia obesity, while maintains adipocyte size constant, suggesting that enhanced pericellular collagen accumulation might constrain adipocyte growth [45].

Recently, Carruthers et al. [46] observed an upregulation of pathways involved in ECM organization in the human type 2 diabetes-specific visceral adipose tissue. Transcriptomic and proteomic data reveal an increase in gene expression and protein abundance of integrins (ITGB1, ITGAM, ITGB2), and thrombospondins (THBS1, THBS4). Furthermore, the CD44 a cell adhesion receptor highly expressed in ECM and extracellular fluids was positively correlated with adipose inflammation and an index of insulin resistance (HOMA-IR) in individuals with diabetes and metabolic syndrome [47]. Endotrophin is the C-terminal cleavage product, involving C5 domain of collagens and has a key role in fibrosis and inflammation [48]. Higher serum endotrophin levels are closely associated with higher glycated hemoglobin (HbA1c) and glucose levels in patients with type 2 diabetes [48]. On the hand, recent studies reported that decreased collagen expression in adipose tissue could directly improve metabolic health in patients with diabetes [3,6].

**Figure 3:**
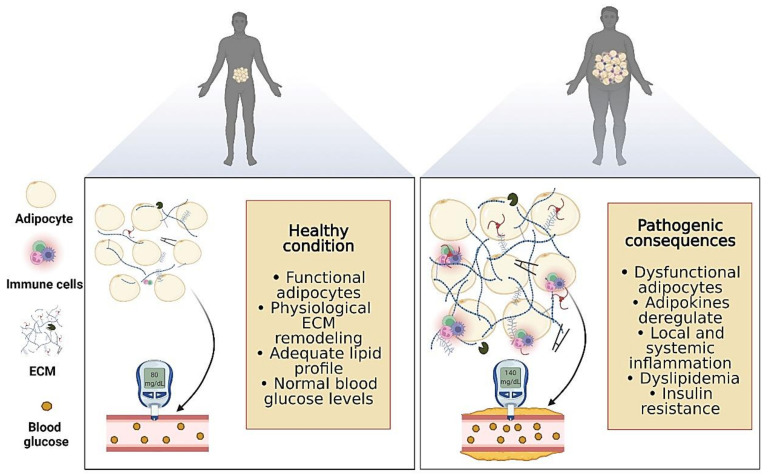
Schematic representation of the healthy condition and pathogenic signals in response to adipose tissue growth during chronic non-transmissible diseases. Biorender web-based software was used to create the figure (License Number PL23TBVZ7M).

Evidence suggests that advanced glycation end-products (AGE), nonenzymatic glycation products, as well as oxidation of proteins and lipids contribute to adipose cellular dysfunction in response to hyperglycemia associated with diabetes [49]. It has been demonstrated that AGE formation is increased in adipose tissue of patients with diabetes when compared to non-diabetic individuals [50]. Moreover, visceral and subcutaneous AGE levels were correlated directly with percentage of HbA1c [51]. Excessive AGE alters adipose tissue function via direct effects on ECM and by binding scavenger receptors and signaling through Rho GTPases, including the DIAPH1 gene, which encodes the diaphanous 1 protein (hDia1) [51]. Strieder-Barboza et al. [51] showed that inhibition of hDia1 attenuated adipocyte glucose uptake. These findings demonstrate that AGE-modification of ECM contributes to adipocyte insulin resistance in human diabetes [51]. Schematic illustration of the pathogenic signals in adipose tissue during chronic non-transmissible diseases is cited in Figure 3.

## 4. Adipose Tissue ECM Structure, Components, and Remodeling

Adipose tissue ECM provides structural support, mechanical stability, elasticity, and adhesion between cells and serves as a reservoir of growth factors and cytokines, essential for adipocyte growth signaling and differentiation [2]. The ECM is produced by adipocytes and stromal cells and consists of a reticular fiber network, whose basic function is to hold the cells in place [4]. The ECM represents a highly dynamic entity responsible for controlling several biological and pathophysiological processes [3]. It is well documented that those physical properties of the ECM microenvironment, such as rigidity, density, porosity, insolubility, and topography (spatial arrangement and orientation) might contribute to the regulation of cell behavior, including cell migration and proliferation and differentiation [2]. The ECM consists of a basement membrane and an interstitial matrix based on chemical and operational traits. The central role of the basement membrane is to provide a physical barrier between the epithelial cells and the connective tissue while still allowing the diffusion of gases and transport of molecules [52]. The interstitial matrix, mainly produced by mesenchymal cells, determines adipocyte dynamics [52]. In particular, components of the ECM include collagens and various classes of adhesion proteins, such as fibronectin, laminin, elastin, and proteoglycans [51].

As the most abundant structural components of the adipose tissue ECM, collagens support tissue architecture and cell functions, as well as morphogenesis [53]. Proteomic studies discovered the presence of 12 collagens in rodent adipose tissue [54,55]. In particular, type I collagen is the main component of the ECM. Distributed throughout the interstitium, it constitutes up to 90% of the total tissue [56]. The fibrils are present at a higher expression in subcutaneous fat when compared to visceral. Hence, the low proportion or percentage of collagen I is responsible for the low stiffness of adipose tissue [57]. However, fibronectin assembly plays a crucial role in maintaining the fibrillar organization of type I collagen [58]. Fibronectin can be cross-linked to an α1(I) collagen chain through factor XIIIa [59]. This molecular communication regulates adipocyte function and cytoskeletal organization [60].

The pericellular ECM is varied, having amounts of collagens I, III, IV, and VI, among other proteins [3]. Collagen IV and laminins are two main elements that self-assemble in the extracellular space to form a network, creating basement membrane ultrastructure in adipose tissue [53]. Nidogen and perlecan bridge the laminin and collagen IV network to preserve basement membrane integrity. The matricellular protein SPARC, expressed in adipose tissue, contributes to basement membrane stabilization [61]. The protein SPARC is overexpressed in the white adipose tissue of obese humans [61]. This mediator of collagen deposition modulates interactions between cells and adipose tissue ECM [61].

Type VI collagen has been shown to interact with other ECM noncollagenous proteins, such as fibulin, lumican, matrilin, and elastin [62]. Elastin is a cross-linked protein that enables tissues to stretch and return to their initial conformation. Elastin expression is decreased in Collagen VI-deficient mice, which have a collagen matrix less permissive to adipocyte hypertrophy [43]. This relationship is crucial in the three-dimensional architecture and impacts intracellular signaling pathways in adipose tissue [43]. The interplay between ECM proteins and nuclear structural framework plays a fundamental role in the transcription steps, as well as adipocyte phenotype switching during their adaptations to the environment [3]. The ECM connects within the fatty tissue through transmembrane proteins such as integrins. The integrins play a central role in cell migration and differentiation and induce genic expression of ECM molecules [63].

Proteoglycans are particular glycoproteins observed at the cell surface in the adipose tissue ECM [64]. They interact with many proteins involved in metabolic homeostasis, fibrosis, and inflammation [64]. Lumican, perlecan, decorin, aggrecan, and biglycan have been either secreted by adipocytes or abundantly present in adipose tissue ECM and fibrosis process [64]. Additionally, proteoglycans contribute to the heterogeneity of the ECM and play an essential role in collagen fibrillogenesis and organization in adipose tissue [64]. Moreover, heparan, chondroitin, and keratan modulate obesity-induced metabolic dysfunction that impacts the progression of obesity-associated morbidities [64].

The modulation of ECM turnover and its components is regulated by key factors that include matrix metalloproteinases (MMPs) and tissue inhibitors of MMPs (TIMPs) [65]. A recent study reported 29 different MMPs whose expression is transcriptionally regulated by stimuli such as immune cells, interleukins, growth factors, and hormones [66]. MMPs are enzymes categorized by substrate specificity and homology into the following six family groups: collagenases (MMP1, -8, and -13), gelatinases (MMP-2 and -9), stromelysins (MMP3, -10, and -11), elastase (MMP-12) matrilysins (MMP-7, 26), membrane-type MMPs (MMP14, -15, -16, -17, -24, and -25), and other MMPs. MMPs are a family of Zn^2+^ or Ca^2+^ enzymes that degrade or remodel the ECM constituents [20,66]. MMPs play an important role in the growth and structural organization, contributing to remodeling through degradation of ECM components and activation of latent growth factors of adipose tissue [20]. The local balance between activated MMPs and TIMPs controls the net result of MMP activity in adipose tissues [65]. However, this balance can be altered in some pathological situations, including obesity and diabetes, inducing an imbalance in MMP activation. Furthermore, MMPs play an essential role in angiogenesis and differentiation of preadipocytes into mature adipocytes, resulting in fatty mass expansion [65,67]. Previous studies showed that multiple MMPs such as MMP-2, MMP-3, MMP-9, MMP-11, MMP-12, MMP-13, and MMP-14 levels are overexpressed in adipose tissue dysfunction [3,57]. Nevertheless, models with higher TIMPs and/or lower MMP activity present protection from disease phenotypes on a high-fat diet (HFD), suggesting that critical molecules tightly modulate fibrotic content and ECM composition.

## 5. Dietary Management and Adipose Tissue ECM Remodeling

### 5.1. High-Fat Diet-Fed Animals Models

Eating behaviors remain the cornerstone in the adipose tissue phenotype. Adipose tissue ECM undergoes remodeling in response to different factors, including weight loss or gain and nutrient excess or deprivation. Specifically, excessive energy consumption, such as HFD, modulates the transcriptional signature of the adipocyte from that of a healthy adipocyte. Previous studies revealed that genes related to fatty acid metabolism and mitochondrial energy transduction are downregulated in animals exposed to HFD. However, genes associated with ECM components, remodeling, cytoskeleton, and inflammation are upregulated, which results in higher collagen deposition, dysfunctional adipose depots, and consequent disruption of metabolic homeostasis.

Recently, Jones et al. [68] evaluated the time-course effects of HFD on adipose tissue ECM remodeling. The study showed more elevated mRNA expression of transforming growth factor-beta (TGF-β) family members, crown-like structures, caspase-3 activity, and collagen 6 in visceral white adipose tissue after 34 weeks of HFD when compared to 8 and 20 weeks, indicating that understanding the role of diet time on ECM remodeling in a time-dependent manner is essential to elucidating adaptive mechanisms. The authors proposed that the transcriptional signature of the pathological adipocytes (34 weeks of HFD) is similar to fibroblastic cells, and pathways responsive to TGF-β are a principal regulator of adipocyte-specific production of ECM, focal adhesions, and cytoskeleton [68]. TGF-β regulates gene expression through several overlapping signaling/transcription factor pathways, including suppressor of Mothers against Decapentaplegic (SMAD), c-Jun N-terminal kinase (JNK), extracellular signal-regulated kinases (ERKs), and transcription factor A/serum response factor (MRTF-A/SRF), which have been considered vital molecular players involved in ECM morphological integrity.

During excessive adipose tissue expansion modulated by HFD, imbalances in proteoglycan synthesis and degradation also lead to fibrosis, one of the hallmarks of adipose tissue dysfunction associated with meta-inflammation and insulin resistance. Biglycan is an ECM protein with a proinflammatory effect highly expressed in adipose tissue in obesity. Adapala et al. [69] demonstrated a decrease in mRNA expression levels of IL-6 and CD68 in the epididymal adipose tissue of biglycan knockout mice, which suggests that target biglycan pathways in adipose tissue may be an effective strategy for suppressing inflammation conditions associated with adipocyte expansion. Like biglycan, lumican interacts with collagens and is associated with repair processes in adipose tissue. Wolff et al. [70] reported that although lumican null female mice exhibited increased mRNA expression levels of collagens (*Col1a1* and *Col3a1*) and *Fn1* in the epididymal adipose tissue, there was decreased insulin sensitivity and liver triglycerides in an HFD-dependent manner. Thus, it is plausible that lumican is part of an adaptive program for the ECM reorganization of the adipose tissue in response to nutrient overload.

MicroRNAs are endogenous small RNAs that post-transcriptionally modulate gene expression with a global impact on eukaryotic proteomes that seem to play regulatory roles in metabolic processes [71]. There is growing evidence of an essential function of miRNAs in regulating the adipose tissue ECM remodeling pathways [5,72,73]. For example, Velazquez et al. [73] suggest that miR155 deletion in an HFD model of diet-induced obesity exacerbates fibrosis and increases collagen 1 expression in mice epididymal adipose tissue. Similarly, Chen et al. [5] showed that miR-181d-regulated metalloproteinase Adamts1 impairs adipogenesis via ECM remodeling. Despite these substantial advances, one individual miRNA may have multiple potential targets, which may coordinate or antagonize adipocyte functions. Moreover, miRNA interactions depend not only on the target sequence but also on the cellular context in which the interactions occur. Therefore, small RNA constitutes an integral part of the puzzle in metabolic diseases, and miRNA-based targeted therapeutics are relevant to a breakthrough in fibrosis treatment.

Body-weight loss decreases pathologies associated with obesity; however, it is challenging to maintain, leading to weight regain or weight cycling [74]. Low-calorie diets are initially successful in this process, but most individuals have low long-term adherence [74]. An elegant study demonstrated an increased expression of MMP-2 and MMP-12, gelatinase activity (MMP-2 and MMP-9) in the rats fed HFD. Interestingly, however, intense MMP activity in adipose tissue can remain during weight loss induced by caloric restriction [75]. The authors suggest that ECM could be less rigid during weight loss and that collagen deposition could be due to robust ECM remodeling and not fibrosis process [75]. In addition, MMP inhibition during weight loss modifies adipose tissue inflammation during weight regain, suggesting that the MMP inhibition could be an exciting target to establish weight cyclers [75]. Those findings may have crucial implications for nutritional therapy and dietary management.

Similarly, Li et al. [76] explain the dichotomous effects of MMP-14 in response to HFD (13 weeks). The authors reported an upregulation of MMP-14 in the established obese adipose tissue leading to enlarged adipocytes in transgenic mice. Furthermore, the mice showed decreased energy expenditure and impaired lipid and glucose metabolism. Mechanistically, the authors found that MMP-14 remodels collagen 6 to produce endorphins, a potent costimulator of collagen accumulation and inflammation. However, when overexpressing MMP14 in the early stage (8 weeks) of HFD, the transgenic mice displayed a healthier metabolic profile, including mitigated fibrosis and inflammation, linked to releasing the mechanical stress to allow ECM remodeling. Thus, understanding the pleiotropic functions of MMPs is vital to comprehend the context and time effects of diet.

Hasegawa et al. [77] identified GTF2IRD1, a member of the TFII-I family of DNA-binding proteins, as a cold-inducible transcription factor that represses adipose tissue fibrosis through PRDM16-EHMT1 (PR domain containing 16—Euchromatic Histone Lysine Methyltransferase 1) complex, which has a potent inducer of the thermogenic phenotype. Adipocyte-selective expression of GTF2IRD1 represses inguinal adipose tissue fibrosis and improves systemic glucose homeostasis independent of body-weight loss in mice exposed to HFD. Furthermore, the authors observed in humans that GTF2IRD1 expression inversely correlates with subcutaneous white adipose tissue fibrosis and visceral adiposity [77]. This study suggests a novel mechanistic insight by which repression of obesity-associated adipose tissue fibrosis through the PRDM16 complex improves systemic glucose homeostasis.

In vitro study indicates that an adjustment in the autophagy process mediates the attenuation of adipose tissue fibrosis to an HFD. Autophagy-related 7 (Atg7) knockout mice showed a significant reduction in collagen deposition in the subcutaneous adipose tissue accompanied by downregulation of the mRNAs encoding three fibrillary collagen alpha chains and other ECM components such as Fn1 (fibronectin 1), Spp1 (secreted phosphoprotein 1 or osteopontin), or ECM modifying factors such as tissue inhibitor of metalloproteinase (TIMP1) and lysyl oxidase-like 1/2 (Loxl1/2). Thus, autophagy inhibition may provide a basis for anti-fibrotic strategies in obesity [78].

Additionally, it has been reported that circadian clock function and its component factors are closely tied into energy metabolism, lipid pathway synthesis, and adipose tissue [79]. The nuclear receptor subfamily 1, group D, member (Nr1d1, formerly called Rev-Erbα) is a core clock component and has been highlighted as a critical link between the clock and metabolism [79]. Under chronic energy excess, Nr1d1 may contribute to metabolic dysfunction and adipose tissue hypertrophy. Adipose-targeted Nr1d1 deletion in mice exposed to HFD showed continued adipose tissue expansion accompanied by a healthier metabolic phenotype with reduced adipose inflammation and fibrosis, as well as preserved systemic insulin sensitivity [79]. This discovery may present a therapeutic opportunity to cope with aberrant ECM in adipose tissue during obesity conditions.

### 5.2. Clinical Trials and Follow up Studies on Adipose Tissue ECM

In clinical studies, the consensus has been that human adipose tissue fibrosis is a consequence of obesity-induced chronic inflammation and hypoxia [80,81], which, in turn, has a strong association with insulin resistance [81]. Different cell types present in adipose tissue fibrosis were identified in obese patients, including T-lymphocytes, mast cells, fibroblastic cells, and CD40+ and CD206+ macrophages [80]. Moreover, previous investigations identified two major pathways for regulating human adipose fibrosis: hypoxia-inducible factor 1-alpha (HIF1α) [82] and TGF-β [83]. In obesity, adipose tissue hypertrophy causes hypoxia that induces activation of HIF1α-dependent gene transcription [84,85]. HIF1α stimulates many extracellular factors, such as collagens and components that establish and remodel the ECM [86]. Similarly, in animal models, levels of TGF-β are highly elevated in both the human blood circulation and adipose tissues in obesity conditions [87]. Consequently, this growth factor plays a significant role in adipose tissue remodeling by inducing ECM protein-coding genes.

Achieving a 5% to 10% weight loss by dietary intervention can produce positive health outcomes in people with overweight or obesity, but long-term weight maintenance has proven to be difficult [88]. Previous research papers investigated a relationship between ECM-related genes and weight regain. In a follow-up study during 5 weeks of low-calorie diet (500 kcal/d) with subsequent 4-week weight-stable diet, a strong correlation was observed between stress and ECM-related genes (*Adamts1*, *Col15a1*, *Col21a1*, *Col5a3*, *Lama3*) in the abdominal subcutaneous adipose tissue [89]. This study suggests that ECM plays a unifying role in body weight fluctuations [89].

In another clinical trial, Roumans et al. [63] investigated the relationship between the expression of ECM genes during weight loss and stabilization and weight regain risk. The results pointed to the relevance of leukocytes in abdominal subcutaneous adipose tissue for the weight regain period. After weight loss, a downregulation of leukocyte integrin genes (*Itga5*, *Itgal*, *Itgam, Itgax*, and *Itgb2*) leads to a higher weight regain risk. Therefore, it is probable that a more significant reduction in ECM remodeling capacity during the weight regain might lead to immune cell trafficking and retention, which suggests that resident inflammation after weight loss increases regain risk and might contribute to worsening of physiologic responses during weight cycling.

Similarly, genetic variation in mRNA levels was studied in obese individuals receiving an 8-week low-calorie diet with a 6-month follow-up [90]. The weight regain risk was increased by the gene variation in *Postn*, *Lamb1*, *Col23a1*, *Fbln5*, and *Fn1*. Thus, weight regulation in overweight and obese individuals may come from cell stress levels generated by inappropriate adipose tissue ECM remodeling during weight loss maintenance [90]. Further studies assessing transcriptomics and proteomic assays are required to confirm these findings in a more extensive cohort study. Furthermore, more investigations are needed to elucidate adjacent molecular pathways or post-translational mechanisms in weight regain states after weight loss. This relevant topic might add new concepts and implications for successful weight management.

**Figure 4:**
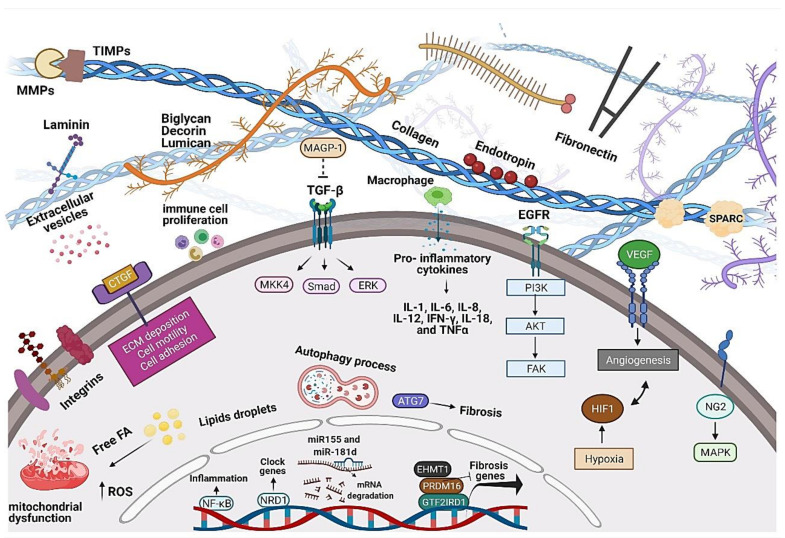
Overview of players and molecular pathways that mediate the progression of adipose tissue fibrosis. Extracellular compartment: collagen, laminin, fibronectin, glycosaminoglycans, proteoglycans (Biglycan, Decorin, Lumican), integrins, growth factors (Tgf-β, Ctgf, and Vegf), enzymes (Mmps and Timps), and vesicles. Intracellular compartment: increase in gene and protein expression linked to ECM components, adipogenesis, cytoskeleton, inflammation (pro-inflammatory cytokines and NfκB), angiogenesis, hypoxia (Hif1), oxidative stress (ROS), clock (Nrd1), and autophagy (Atg7). Biorender web-based software was used to create the figure (License Number ND23TBWD1P).

Therefore, intermittent fasting shows promise as a primary care intervention for adipose tissue ECM remodeling, but little is known about long-term sustainability and health effects. Longer-duration investigations are needed to understand how intermittent fasting might contribute to effective weight-loss strategies and adipose tissue fibrosis. From a clinical point of view, dietary changes should be individualized, tailored to food preferences, and allow for flexible approaches to reducing calorie intake, increasing the motivation and compliance of overweight and obese patients. Potential adverse effects of very-low-calorie or fat diets in health outcomes must be considered. A summary of leading players and molecular pathways that mediate adipose tissue fibrosis progression in rodents and humans is reported in Figure 4.

## 6. The Emerging Role of Exercise Training on Adipose Tissue ECM Remodeling

### 6.1. Animal Models

An emerging body of evidence suggests that exercise training is a potent mediator of lipolysis, preventing fat accumulation and attenuating rapid ECM expansion. Some reports indicate that exercise training would be a promising non-pharmacological strategy for maintaining lipid and glucose metabolism and influencing the release of adipokines in the adipose tissue. The protective effects of exercise training in the background of more robust adipose tissue expansion and energy surplus may shed light on its ability to regulate ECM remodeling. The first evidence describing the beneficial effects of exercise on adipose tissue ECM remodeling was shown seven years ago. Kawanish et al. [16] verified that treadmill training (60 min/day at 15–20 m/min, 5 times/week for 16 weeks) attenuates collagen deposition (collagen 1 and 3α) and fibroblast activation. It also reduces macrophage infiltration in visceral adipose tissue of mice exposed to HF diet and downregulates *Tgf-β* and *Timp-1* mRNA levels. These findings support the hypothesis that aerobic exercise training leads to adipose tissue ECM remodeling, which regulates fibrosis by macrophage mitigation in obese mice. This investigation may contribute to developing new therapeutic strategies against deleterious alterations resulting from obesity.

More recently, Li et al. [7] provided plausible mechanisms for the beneficial effects of exercise on adipose tissue fibrosis in obesity. Aerobic exercise training (50 min/day at 75% maximum oxygen consumption, 5 times/week for 12 weeks) attenuated the increased fibrosis-related gene expression (*Col3α1*, *Col6α1*, *Lox,* and *Fibronectin*) in epididymal adipose tissue of mice exposed to HFD for 12 weeks. Additionally, exercise increased peroxisome proliferator-activated receptor gamma (PPARγ), which has been shown to inhibit adipose tissue fibrosis, and decreased hypoxia-inducible factor 1-alpha (HIF1α) expression. These effects clarify that PPARγ may downregulate HIF1α and promote angiogenesis as well as the lipolytic, anti-inflammatory process, which contributes to anti-fibrosis effects. Furthermore, the authors hypothesize that attenuation of the progression of fatty tissue fibrosis by exercise contributes to a preventive development in the HFD-induced disturbance of glucose metabolism. Hence, a mechanistic view of fibrosis could provide essential clues for health progress and clinical practice for type-2 diabetes and obesity patients.

In other study, Javaid et al. [91] found that upregulated mRNA expression levels of TGF-β1 and COL1A1 in HFD mice were significantly downregulated by exercise (8 weeks of treadmill running during their active cycle, 5 days/week) alone and were further suppressed on combining exercise and diet (normal chow diet). Interestingly, exercise exerts its anti-fibrotic action by suppressing caspase 1, as well as NOD-like receptor family 3 (NLRP3) inflammasome components and its stimulatory factors such as thioredoxin-interacting protein (TXNIP) and p62.

In an elegant study, we demonstrated that paternal resistance training (RT) (8 weeks, 3 times per week with progressive overload (eight climbs, two sets with each load of 50%, 75%, 90%, and 100% of the maximum load capacity)) compensated for the detrimental effects of HFD in the male offspring preventing the upregulation of genes linked to adipose tissue fibrosis (*Ctgf, Vegf, Cebpa, Srebp1, Mcp1,* and *Nfκb*), pro-inflammatory cytokines, MMP-2 activity, ROS production, as well as collagen 1 deposition in epidydimal adipose tissue [92]. These discoveries accompanied increased antioxidant enzymes while decreasing pro-oxidant agents, MMP-2/9 activity, and metabolic markers (insulin, leptin) in the bloodstream. Interestingly, new findings reveal that the possible interplay among ECM remodeling, inflammation, and oxidative stress can control the healthy adipose tissue phenotype in the intergenerational exercise model [92]. Paternal lifestyle before gestation might partially prevent the obese phenotype of first offspring [92].

In contrast with previous studies, Pincu et al. [93] showed that HFD induced collagen accumulation and substantially increased gene expression of ECM proteins, including MMPs, TIMPs, and COL1A in epididymal adipose tissue. Still, exercise training (10–17 m/min, 60 min, 5 times/week for 16 weeks) did not alter mRNA levels. The authors suggested that the combination of animals aged at the final experiment (28 wks) and an extremely high-fat diet (60% of the calories from fat) might curtail the beneficial effects of exercise training. However, this previous result should be interpreted with caution because the authors used a small sample size (3 mice in the HFD exercise group), limiting the extrapolation of findings. Furthermore, the authors analyzed only gene expression, restricting molecular interpretations. In our view, functional, morphological, molecular, and cellular responses might help furnish a complete picture of the ECM adaptation in response to exercise.

In human and rodent investigations, aging is associated with increased visceral adipose tissue and inflammation [94,95,96], potentially leading to the establishment of fibrosis. Intriguingly, Ziegler et al. [97] showed that epididymal adipose tissue fibrosis and Tgf-β mRNA levels remained unchanged in response to voluntary wheel running (10 weeks with resistance fixed at 1.5 g throughout the intervention) or voluntary wheel running with progressive resistance (5 g in week 1, 6 g in week 2, and then increased 1 g every second week ending at 10 g in week 9–10) in adult and old mice. Although Tgf-β constitutes an integral part of the puzzle [12,53,68], we should be open to the possibility that other regulatory mechanisms exist. The pleiotropic effects of exercise and the complexity of responses at a molecular level in current literature suggest no distinct pathway mediating beneficial exercise on adipose tissue ECM. Thus, it is essential to emphasize that cellular homeostasis is achieved by a delicate balance between multiple pathways and players required to carry out complex physiological processes.

Strong evidence revealed that an imbalance in sex hormones causes a profound impact on adipose tissue morphology, which compromises ECM function and consequently triggers metabolic dysfunction [98]. Acknowledging this, Duarte et al. [98] investigated whether resistance training (RT) (10 weeks, 3 times per week with progressive overload (four climbs, one set with load of 65, 85, 95, and 100% of the maximum load capacity) may be an alternative strategy to combat the harmful effects promoted by estrogen decay through modulation in gene expression patterns in the ECM of visceral adipose tissue of ovariectomized rats. The authors revealed that the lack of estrogen increased the visceral, parametrial, and subcutaneous adipocyte areas. The ovariectomy model also upregulated *Mmp-2*, *Mmp-9*, *Tgf-β*, *Ctgf*, and *Vegf-α* mRNA levels and MMP-2 activity. On the other hand, RT decreased the adipocyte area of three fat depots (visceral, parametrial, and subcutaneous), downregulated *Mmp-9*, *Tgf-β*, and *Ctgf* gene expression, and reduced MMP-2 activity. These data suggest that RT may play an essential role in maintaining and remodeling ECM, previously altered by a lack of estrogen, promoting an improvement in adipose tissue homeostasis. Therefore, ECM adipose function will have high clinical significance in menopausal conditions.

Clinical trials that evaluate the effects of abrupt estrogen deficiency in women undergoing oophorectomy on genes expression and proteins involved in adipocyte expansion and function (ERα, PPARγ2, C/EBPα, aromatase, adiponectin, and LPL), extracellular matrix remodeling and fibrosis (COL6(a1, a2, a3), COL4a1, and TGFβ), and inflammation (IL-6 and TNFα) are interesting to clarify the plausible mechanisms observed in animal studies.

Those involved with the prescription of RT (8 weeks, 3 times per week), including coaches, rehabilitation specialists, and exercise physiologists, must understand the acute program variables to potentiate training adaptations. Our laboratory found no difference for pro and intermediate MMP-2 activity in visceral adipose tissue between lower volume RT (four climbs, one set with load of 50%, 75%, 90%, and 100% of the maximum load capacity) and sedentary group. In contrast, higher volume (eight climbs, two sets with each load of 50%, 75%, 90%, and 100% of the maximum load capacity) induced lower active MMP-2 activity in healthy rats, contributing to a delay in the adipogenesis process [99]. These findings suggest that adipose tissue ECM remodeling is modulated by exercise volume and may be a key element in adaptive responses. This exercise protocol should receive special attention as it could be crucial for rehabilitation and open new avenues for therapeutic interventions in humans. However, future studies are needed to examine other MMP families and TIMPs involved in the fibrosis process.

We noted that exercise training presents significant discrepancies between protocols. However, it can be determined that treadmill training and strength exercise are organized and effective modalities, managing important acute exercise variables. Each exercise type presents advantages and disadvantages, but it is vital to highlight that no modality included all the various kinds of adipose tissue and depots. In addition, transgenic rodents with target underexpression into the genome can help identify new and complex mechanisms in adipose tissue ECM in response to exercise.

It is important to emphasize that prior studies only evaluated the quantity of collagen deposition; however, the structure or quality may be necessary when assessing tissue fibrosis. Collagen quality (structure or stiffness) can clarify tensile strength, cell adhesion, chemotaxis, and migration, consequently modulating tissue development [12,56]. Moreover, it is unknown whether different exercise protocols modulate laminin, elastin, and proteoglycans. Some evidence suggests heterogeneous adipokine levels in different adipose depots after exercise training [25,100], possibly interfering with the process of adipose tissue fibrosis. Hence, future investigations could evaluate the specific molecular signatures in depot-specific fibrosis. Studies to date have usually assumed single molecules within an individual function. Novel biomarkers involved in the interplay between adipose tissue and other organs, or pathways should be investigated as they might provide new insights for health-related outcomes.

### 6.2. Human Studies

In a clinical study, Zaidi et al. [18] investigated the effects of long-term combined exercise (60 min/day, 2 times/week for 12 months); aerobic (interval running/step training and spinning (rated perceived exertion ≥15; 5–15 min duration) and resistance training (free weights for large muscle groups at moderate intensity) on the remodeling markers MMP-9, TIMP-1, protein extracellular matrix metalloproteinase inducer (EMMPRIN), and galectin-3 in the subcutaneous adipose tissue of patients with type 2 diabetes mellitus and coronary artery disease. The exercise group displayed higher EMMPRIN mRNA levels in subcutaneous adipose tissue post-exercise training period. This marker also increased when compared to the control group, which indicates that people with the longstanding disease need higher training volumes or intensity, as well as dietary interventions, to respond to the exercise protocol. These authors have not accounted for physical activity levels in the control group. Moreover, they did not address the dietary intake and period between the last exercise session and subcutaneous adipose tissue withdrawal, which obscure the chronic effects of exercise on ECM remodeling.

In another study, Nankam et al. [17] measured the regional changes in transcriptome signatures between abdominal (aSAT) and gluteal subcutaneous adipose tissue (gSAT) in obese black South African women. They evaluated 12-week combined exercise training (aerobic and resistance training (60–70% heart rate peak) progressing from 40 to 60 min, 4 days per week) in terms of mRNA levels in a depot-specific manner. The biopsies were collected at least 72 h after the last exercise training to exclude the potential acute effects of the session. The authors observed an increase in *Mmp9*, *Col1a1*, and *Col6a2* in gSAT and *Fndc1* and *Col1a1* in aSAT mRNA levels in response to exercise training. The authors explain that proinflammatory signaling in adipocytes mediates ECM modeling, and the reduction of stored triglycerides in response to exercise training might precede an extensive local ECM remodeling.

In a narrative review, De Bari et al. [6] reported that epidemiological investigations differ substantially depending on the experimental design and demographic aspects. The impacts of gender, race, and several comorbidities appear to have a considerable influence on adipose tissue fibrosis in clinical studies. More studies are essential to advance how patient demographics alter adipose tissue fibrosis after exercise interventions. Overall, fatty tissue fibrosis is not routinely screened in clinical practice; however, it can profoundly affect patient outcomes and therapeutic options.

More recently, Silva et al. [101] evaluated the effect of concurrent training (12 weeks, 3 times per week) in the ECM of SAT in people living with HIV/AIDS. They observed that concurrent training effectively improved aerobic and strength performances, promoting a decrease in the size heterogeneity of adipocytes and elastic fiber deposition, improving the SAT fibrosis profile. These results have clinical importance for the overall health and longevity of these patients since HIV infection is associated with the pathophysiology of adipose tissue alterations, contributing to the onset of metabolic alterations and the risk of cardiovascular disease [15,102,103].

Two commonly used exercise training prescriptions (12 weeks (4 days week–1) of either moderate-intensity continuous training (MICT; 70% maximal heart rate, 45 min) or high-intensity interval training (HIIT; 90% maximal heart rate, 10 × 1 min) increased collagen type 5a3, while reduced MMP-9 and fat cell size in human subcutaneous adipose tissue of adults with obesity [104]. However, these exercise-mediated changes did not induce weight loss. These data suggest that the impact of exercise on ECM adipose tissue and the overall health of humans are far from linear and point to a complex set of interactions. Thus, a long-term multifactor approach (cognitive-behavioral therapies, individual diet, exercise, and medical intervention) can be essential for weight loss.

**Figure 5:**
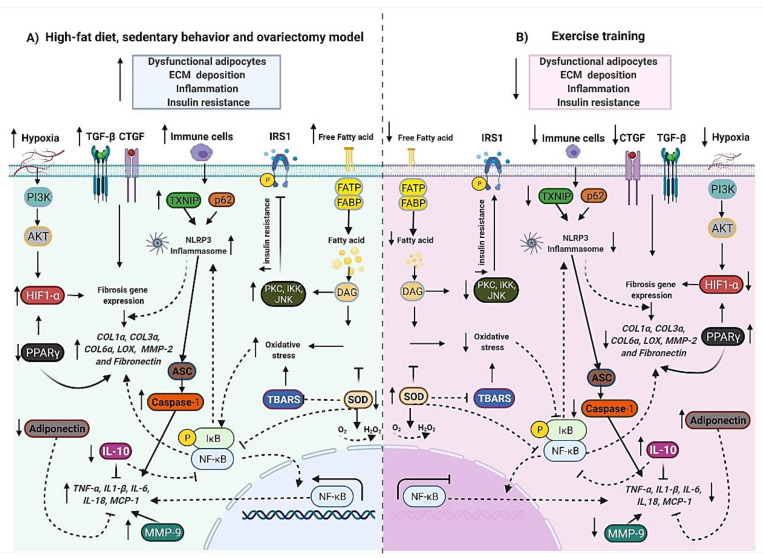
The molecular landscape of adipose tissue extracellular matrix remodeling in response to dietary patterns and exercise training. (**A**) The high-fat diet, sedentary behavior, and ovariectomy model induces dysfunctional adipocytes (↑ lipid accumulation), ECM deposition (↑ pro-fibrotic gene expression), inflammation (↑ NLRP3 inflammasome, NF-κB, pro-inflammatory cytokines, macrophage and MMP-9), oxidative stress (↑ ROS production and hiobarbituric acid reactive substances (TBARS)), and insulin resistance (↑ insulin and blood glucose levels). (**B**) Exercise attenuates dysfunctional adipocytes (↓ lipid accumulation), ECM deposition (↓ pro-fibrotic gene expression), inflammation (↓ NLRP3 inflammasome, NF-κB, pro-inflammatory cytokines, macrophage, and MMP-9), oxidative stress (↓ ROS production and hiobarbituric acid reactive substances (TBARS)), ↑ antioxidant activity (superoxide dismutase (SOD)) and insulin resistance (↓ insulin and blood glucose levels). Biorender web-based software was used to create the figure (License Number SQ23TBWSCJ).

Investigations involving different exercise frequency, time, and intensity could elucidate the potential mechanisms involved in ECM plasticity to protect from obesity conditions and other non-transmissible chronic conditions. We suggest further randomized controlled trials comparing exercise and dietary changes in different populations, with comparisons between RT versus endurance training and concurrent training (combination of endurance training and RT), as exercise modality may affect molecular pathways. It is essential to highlight that the variability between studies and heterogeneity of exercise protocols limit the understanding of the dose-response effect of exercise. There is a need for further research to understand the impact of interrupting exercise and the influence of lifelong exercise, not just for a specific time. For innovative molecular approaches, a combination of lipidomics with transcriptomics and proteomics post-exercise can be essential for discovering a new regulatory mechanism in adipose tissue fibrosis. To better demonstrate our results, we have summarized the molecular landscape of adipose tissue ECM in response to dietary patterns, chronic diseases, and exercise training in Figure 5.

## 7. The Potential New Pharmacological Approaches to Attenuate Adipose Tissue Fibrosis Based on Positive Exercise Effects

The emerging beneficial role of exercise training on adipose tissue ECM remodeling adds a level of complexity to any discussion of drugs/compounds to mimic or potentiate the chronic effects of exercise. Indeed, the poor prognosis in patients with adipose dysfunction emphasizes the need for developing novel pathways for testing new drugs, beyond metabolic modulation approaches. Based on the studies summarized in this review, the drugs development must thus necessarily focus on novel approaches to inhibition of growth factors (TGF-β, CTGF and VEGF), hypoxia inducible factor (HIF1α), and inflammatory factors (NLRP3 inflammasome, NF-κB, pro-inflammatory cytokines and macrophage). Another potential target for new drugs may be the activation of several antioxidants signaling pathways (nuclear factor erythroid 2-related factor 2, glutathione peroxidase, SOD, and catalase) that are found to be associated with oxidative stress and show a protective effect against stressors by increasing the release of several cytoprotective enzymes and also exert anti-inflammatory effect against adipose tissue fibrosis [92].

PPARγ activation following exercise has been proven to downregulate HIF-1α expression and reduce adipose tissue collagen deposition, which is essential for retarding ongoing adipose tissue fibrosis [7]. Furthermore, PPARγ agonist treatment primes monocytes into anti-inflammatory M2 macrophages and reduces proinflammatory cytokine expression [105]. In parallel, PPARγ selectively induces lipogenesis in the adipose tissues, leading to lowered circulating triglycerides and free fatty acids, and insulin resistance [106]. Hence, PPAR agonists with minimal off-target effects, high selectivity, and bioavailability open a new repertory for clinical medicine.

In our laboratory, we found that paternal exercise upregulated adiponectin levels in offspring white adipose tissue exposed to HFD, which may have contributed to anti-fibrotic and anti-inflammatory effects [25]. In support of our evidence, a transgenic mouse model with elevated circulating adiponectin levels has a dramatically improved systemic insulin sensitivity, reduced age-related tissue inflammation and tissue fibrosis, and a prolonged healthspan and median lifespan [107]. Possibly, a more prominent ECM can lead to a further reduction in beneficial adipokines, such as adiponectin. These findings identify a novel function for this pleiotropic adipokine in regulation of tissue remodeling. Restoring adiponectin signaling in patients with tissue fibrosis might represent an innovative pharmacological strategy for adipose tissue.

Additionally, it has been demonstrated that alternatively activated macrophages (M2-like) participate in extracellular collagen degradation through mannose receptor phagocytic pathway [108], suggesting there is an interplay between autophagy and inflammation cascade activation during the progress of adipose tissue fibrosis. Considering that exercise potentially suppresses caspase 1 and NLRP3 inflammasome system in the adipose tissue fibrosis model [91], molecules that interfere in the various steps of the autophagic machinery can hopefully open new possibilities for effective drug development in fibrosis treatment.

The dynamics of fibrosis are regulated by MMPs, which is a protein family that cleaves ECM proteins, enabling remodeling. The balance between MMPs and TIMPs must be well managed to optimize positive exercise effects. Recently it was demonstrated that treatment with MMP inhibitors based on protein therapeutics with low toxicity, including antibodies and TIMPs offer an excellent potential for higher selectivity due to the large antigen–antibody, or TIMP-MMP protein–protein interaction surface [109]. Thus, developing novel MMP inhibitors drugs accompanied by optimal delivery can be taken into consideration.

## 8. Conclusions and Perspectives

The extracellular matrix in adipose tissue is not only a simple morphological structure but provides a biochemical and biomechanical context in adipocyte dynamic behavior, modulating numerous physiological and pathophysiological processes. The studies summarized in this review show that a high-calorie diet results in substantial adipose tissue ECM remodeling, leading to fibrosis and the onset of tissue dysfunction. However, in conjunction with a nutritionally balanced diet, exercise training is a remarkable potential strategy for lipolytic activity, preventing rapid ECM expansion in different adipose tissue depots. Despite the different exercise protocols reversing the progression of adipose tissue fibrosis in animal models, the beneficial impacts on humans remain a controversial topic. The lack of detailed understanding of fatty tissue architecture, function, and molecular players remains a challenge for further studies.

Understanding the complex crosstalk between ECM proteins (collagens, laminin, integrins, fibronectin, glycosaminoglycans, proteoglycans), growth factors (Tgf-β, Ctgf, and Vegf), remodeling enzymes (MMPs and TIMPs), adipocyte function (PPARγ), inflammation cascade (NLRP3 inflammasome, NF-κB, pro-inflammatory cytokines, and macrophage), hypoxia (Hif1), oxidative stress, circadian clock (Nrd1), and autophagy (Atg7) is essential for developing new therapeutic interventions for chronic diseases and novel strategies for tissue engineering and regenerative medicine. Defining these molecular pathways and specific mechanisms that mediates the positive effects of exercise and diet intervention on adipose tissue fibrosis are critical factors to improve health and clinical outcomes.

Since the rate of metabolic diseases and obesity is increasing globally, we must better understand the fibrotic causes and their complex mechanisms to create optimal treatments. Constituents of the ECM environment may likely provide possible pharmacological and non-pharmacological intervention targets. Furthermore, a better knowledge of the forces that drive the anti-fibrotic effect in adipose tissue promises to be a productive area of exercise physiology and sports nutrition research.
